# The Role of Tc-99m DTPA Renal Dynamic Scintigraphy in Retroperitoneal Liposarcoma

**DOI:** 10.1155/2020/9765162

**Published:** 2020-02-22

**Authors:** Ying Wang, Ming Li, Shundong Dai, Yaming Li

**Affiliations:** ^1^Department of Nuclear Medicine, First Hospital of China Medical University, Shenyang, Liaoning, China; ^2^Department of Urology, Shengjing Hospital of China Medical University, Shenyang, Liaoning, China; ^3^Department of Pathology, First Hospital of China Medical University, Shenyang, Liaoning, China

## Abstract

**Purpose:**

Technetium-99m diethylene triamine pentaacetic acid (Tc-99m DTPA) renal dynamic scintigraphy is a widely used imaging technique that evaluates renal function of patients with extrarenal abnormalities, but its clinical value in potentially offering us information on proliferation of liposarcoma has not yet been reported.

**Methods:**

We retrospectively reviewed 7 patients with histopathologically confirmed retroperitoneal liposarcoma who underwent Tc-99m DTPA renal dynamic scintigraphy. The clinical data, histopathological findings, Glomerular Filtration Rate (GFR), and Tc-99m DTPA uptake were recorded.

**Results:**

Dedifferentiated liposarcoma and well-differentiated liposarcoma showed dissimilar degrees of Tc-99m DTPA uptake, an observation that correlated with Ki-67 expression (*p* < 0.01). 4 of the 7 patients were diagnosed with dedifferentiated liposarcoma, showing a moderate uptake of Tc-99m DTPA and greater than 20% Ki-67 expression on histological slides. Meanwhile, the remaining 3 patients, diagnosed with well-differentiated liposarcoma, showed no uptake of Tc-99m DTPA and Ki-67 expression of less than 5%.

**Conclusions:**

This study suggests that Tc-99m DTPA renal dynamic scintigraphy provides diagnostic value in patients with retroperitoneal liposarcoma, not only in evaluating renal function but also in visualizing lesion-related radionuclide uptake, which may potentially offer further clinical insights into tumor proliferation and provide prognostic value for clinical outcomes in patients with retroperitoneal liposarcoma.

## 1. Introduction

Tc-99m diethylenetriaminepentaacetic acid (DTPA) undergoes glomerular filtration but neither renal tubular secretion nor reabsorption. Tc-99m DTPA renal dynamic scintigraphy becomes a useful tool for clinicians in assessing renal function, especially for patients with suspected renal impairment. Nevertheless, Tc-99m DTPA renal dynamic scintigraphy is also valuable in examining extrarenal abnormalities through the uptake of radionuclide by lesions. Despite this, studies on the extrarenal uptake of Tc-99m DTPA have been limited to retroperitoneal abscesses [1], “phantom kidney” phenomena, and some tumors, including hypervascular tumors [2], aortic aneurysms [3], extramedullary plasmacytoma [4], and hepatic haemangioma [5]. Goshen et al. [6] also presented the use of Tc-99m DTPA renal scintigraphy to find palpable soft tissue tumors at any location in the body and noticed a chordoma through the uptake of Tc-99m DTPA. However, the uptake of Tc-99m DTPA in retroperitoneal liposarcoma has not been described previously. In clinics, we noticed a phenomenon that well-differentiated liposarcoma and dedifferentiated liposarcoma have different degrees of uptake of Tc-99m DTPA in renal dynamic scintigraphy, which may provide us some information on the pathology of the tumor and hence, we discuss here.

## 2. Patients and Methods

We conducted studies on human participants according to the guidelines of the Institutional Patient Care and Use Committee of the First Hospital of China Medical University. The patients of this retrospective study were selected between 1st January, 2012, to 30th June, 2014, from the first hospital of China Medical School. Patient selection was based on the following inclusion criteria: (1) the patient underwent Tc-99m DTPA renal dynamic scintigraphy to assess the renal function; (2) liposarcoma was histopathologically confirmed; and (3) complete clinical data for the patients were available. The study contained 7 patients (5 female and 2 male patients; age range, 52–73 years; mean age, 59.6 years). Because this was a retrospective study, informed consent was not needed.

All of the patients received a 185MBq (5mCi) bolus injection of Tc-99m DTPA while in the supine position. Renal dynamic scintigraphy was acquired on a SPECT/CT scanner (Siemens Symbia-T2 TruePoint) with a low-energy collimator, a 128 × 128 matrix, and 20% energy window after dynamic scanning for sixty minutes, The Glomerular Filtration Rate (GFR) was calculated.

*Grading Criteria of Accumulation*. The uptake of extrarenal abnormalities was graded by consensus agreement of two radiologists in the renal parenchyma phase according to the scale as follows [7]:

  No uptake: the activity was the same as that of the background
  Mild uptake: the activity was higher than that of the background but lower than that of the spleen
  Moderate uptake: the activity was equal to or higher than that of the spleen but lower than that of the kidney
  Severe uptake: the activity was equal to that of the kidney
  Criteria of GFR: normal: GFR ≥ 40 ml/min

All the lesions were surgically removed. The pathological results were diagnosed as liposarcoma, and then, the Ki-67 expression of the tumors was evaluated.

## 3. Statistical Analysis

The Mann–Whitney *U* test was used to estimate the correlation between the degrees of Tc-99m DTPA uptake and Ki-67 expression in the tumor. It was also used to estimate the correlation between the uptake of the Tc-99m DTPA in the tumor and the function of the kidneys. Statistical significance was determined as *p* values less than 0.05. We used SPSS (version 23.0) for all statistical analyses.

## 4. Results

Seven patients with retroperitoneal liposarcoma underwent Tc-99m DTPA renal dynamic scintigraphy, in which 4 patients showed moderate uptake of Tc-99m DTPA and the other 3 patients showed no uptake of Tc-99m DTPA in the lesions. The clinicopathological data are listed in Table 1.

In our study, the left renal GFR of one patient with lesions obviously impinging the left kidney decreased deeply and the bilateral renal GFR of two patients with lesions slightly impinging the adjacent kidney decreased slightly. However, these three patients showed normal creatinine and blood urea nitrogen. The other four patients with lesions far away from the kidney revealed normal function in both kidneys.

**Figure 1:**
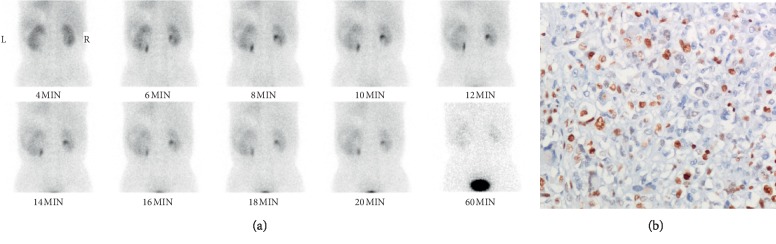
A 73-year-old man with dedifferentiated liposarcoma in the left retroperitoneum. (a) The renal parenchyma phase showed moderate uptake of Tc-99m DTPA, TGFR = 66.1 ml/min, LGFR = 34.0 ml/min, and RGFR = 32.1 ml/min. Serum renal function was normal. (b) Ki-67 expression is 50% (×200).

**Figure 2:**
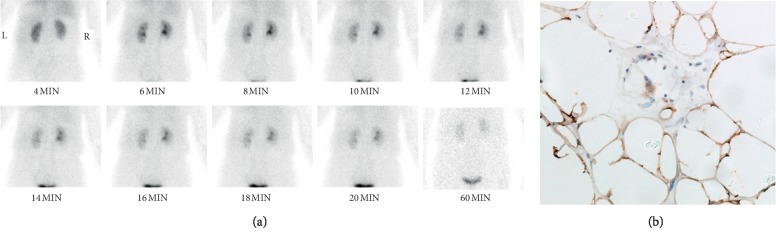
A 64-year-old woman with well-differentiated liposarcoma in the right retroperitoneum. (a) The renal parenchyma phase shows no uptake of Tc-99m DTPA, TGFR = 67.6 ml/min, LGFR = 34.2 ml/min, and RGFR = 33.4 ml/min. Serum renal function was normal. (b) Ki-67 expression is less than 5% (×200).

We also found a phenomenon that dedifferentiated liposarcoma and well-differentiated liposarcoma showed different degrees of uptake of Tc-99m DTPA, which were correlated with Ki-67 expression of the imaged tumors (*p* < 0.01). 4 of 7 patients were diagnosed as dedifferentiated liposarcoma, showing moderate uptake of Tc-99m DTPA (shown in Figure 1(a)), and Ki-67 expression was greater than 20% (shown in Figure 1(b)). Meanwhile, the remaining 3 patients were diagnosed as well-differentiated liposarcoma, showing no uptake of Tc-99m DTPA (shown in Figure 2(a)), and Ki-67 expression was less than 5% (show in Figure 2(b)). Furthermore, we did not find any relationship between the uptake of Tc-99m DTPA in the tumor and the function of the kidney(s) (*p* > 0.05).

## 5. Discussion

Liposarcoma is the second most common soft tissue sarcoma, accounting for up to 15% of adult soft tissue sarcoma. The peak incidence is aged 50–70 years [8]. According to the World Health Organization and others, liposarcoma is currently subclassified into three separate biologic groups encompassing five subtypes including (1) well-differentiated liposarcoma and dedifferentiated liposarcoma, (2) myxoid and round cell liposarcoma, and (3) pleomorphic liposarcoma. Each group is characterized by specific genetic alterations presumed to drive tumor initiation [9].

By far, the most common liposarcoma subtypes in the retroperitoneumin are the well-differentiated liposarcoma and dedifferentiated liposarcoma, representing over 60% of all liposarcoma [10]. Other histological categories including myxoid, round cell, and pleomorphic liposarcoma occur predominantly in the extremity and are rare or “nonexisting” in the retroperitoneum [9, 11–13].

The concept of dedifferentiated liposarcoma was introduced by Evans [14] as “tumor containing well-differentiated liposarcoma and cellular nonlipogenic spindle cell or pleomorphic sarcoma.” Dedifferentiated liposarcoma is usually an abrupt transition from well-differentiated liposarcoma to a region of nonlipogenic sarcoma. Histological subtype of liposarcoma is very important during the process of disease. Dedifferentiated liposarcoma is high-grade, aggressive tumor with a systemic metastatic rate of 5% to 20% [15] and poor prognosis [16], whereas well-differentiated liposarcoma is low-grade tumor with a more indolent biological behaviour and progress more slowly. Five-year disease-specific survival in patients with dedifferentiated liposarcoma is 44%, compared to 93% in patients diagnosed with pure well-differentiated liposarcoma [17, 18].

Liposarcomas usually present as a slow-growing, painless mass [19]. Symptoms occur when it is huge enough to impinge the adjacent structures or local invasion [20] and influence the function of adjacent organs like kidney. So it is necessary to evaluate the renal function preoperatively.

In our study, the left renal GFR of one patient with lesions obviously impinging the left kidney decreased deeply and the bilateral renal GFR of two patients with lesions slightly impinging the adjacent kidney decreased slightly. However, these three patients showed normal creatinine and blood urea nitrogen, which may be due to powerful compensatory function of kidneys. So it is meaningful to use Tc-99m DTPA renal dynamic scintigraphy to evaluate the unilateral kidney of patients with abdominal lesions. The other four patients with lesions far away from the kidney revealed normal function of each kidney.

Importantly, we noticed a phenomenon that dedifferentiated liposarcoma and well-differentiated liposarcoma showed different degrees of uptake of Tc-99m DTPA. The dedifferentiated liposarcoma showed moderate uptake of Tc-99m DTPA, whereas the well-differentiated liposarcoma showed no uptake. As we know, dedifferentiated liposarcoma is associated with worse outcomes compared with well-differentiated liposarcoma, so Tc-99m DTPA renal dynamic scintigraphy may provide us some outcome information beyond kidney function. An even more interesting thing is that the different degrees of uptake of Tc-99m DTPA were correlated with Ki-67 expression of the tumors (*p* < 0.01). Four patients with dedifferentiated liposarcomas showed moderate uptake of Tc-99m DTPA, in which Ki-67 expression were almost greater than 20%. The remaining three patients with well-differentiated liposarcomas showed no uptake of Tc-99m DTPA, while the Ki-67 expression was less than 5%.

Ki-67 antigen named as antiproliferative nucleoprotein monoclonal antibody is a kind of hyperplasia of cell cycle-related nuclei and mainly expressed in proliferating cells. It is considered as a gold standard for accurately reflecting the cell proliferation activity [21] and is usually used to evaluate the biology behaviour of the tumor [22]. Its expression is usually associated with clinical outcome in several tumors, such as lung, breast, hepatocellular, and soft tissue carcinoma [23]. Another study also showed that Ki-67 has very good sensitivity and specificity in lipotumors, especially in dedifferentiated liposarcoma [24]. Our study also indicated that Ki-67 expression of dedifferentiated liposarcoma is greater than that of well-differentiated liposarcoma. The exact mechanism of Tc-99m DTPA uptake is still not clear; it may be due to the increased vascularity, prolonged retention of radionuclide, or the accumulation of the lesion. Furthermore, in our study, we did not find any relationship between the uptake of the radiopharmaceuticals in the tumor and the function of the kidneys (*p* > 0.05).

Our study has some limitations. A small number of patients and pathological types were included in our study. Future studies should include a larger sample and all the pathological types of liposarcoma and potentially even other extrarenal abnormalities. Further evaluation of the relationship between the Tc-99m DTPA uptake and patient's outcome is also needed.

## 6. Conclusion

Our study recommends that clinical use of Tc-99m DTPA renal dynamic scintigraphy in patients with retroperitoneal liposarcoma is valuable not only in evaluating renal function but also in providing a scintigraphic record of lesion-related radionuclide uptake, which may potentially provide information on tumor proliferation to clinicians and predict the clinical outcomes in patients with liposarcoma.

## Figures and Tables

**Table 1:** Clinicopathologic data of 7 patients with liposarcoma who underwent Tc-99m DTPA renal dynamic scintigraphy.

Patient no.	Sex	Age (yr)	Cr	BUN	Side of tumor	Impinge the kidney	GFR (ml/min)	Uptake of Tc-99m DTPA	Pathological pattern	Expression of Ki-67 (%)
1	Female	53	Normal	Normal	Median and left	No	L = 44.3 R = 43.1	Moderate	Dedifferentiated liposarcoma	+50
2	Female	65	Normal	Normal	Right	No	L = 51.9 R = 46.5	Moderate	Dedifferentiated liposarcoma	+30
3	Female	52	Normal	Normal	Left	Yes (obvious)	L = 18.6 R = 41.0	Moderate	Dedifferentiated liposarcoma	+20
4	Male	73	Normal	Normal	Left	Yes (slight)	L = 34.0 R = 32.1	Moderate	Dedifferentiated liposarcoma	+50
5	Female	53	Normal	Normal	Right	No	L = 51.8 R = 46.6	No	Well-differentiated liposarcoma	+2
6	Male	57	Normal	Normal	Median and left	No	L = 54.8 R = 67.6	No	Well-differentiated liposarcoma	0–1
7	Female	64	Normal	Normal	Right	Yes (slight)	L = 34.2 R = 33.4	No	Well-differentiated liposarcoma	<5

Cr indicates creatinine; BUN indicates blood urea nitrogen; GFR indicates glomerular filtration rate; L indicates left; R indicates right.

## Data Availability

The data used to support the findings of this study are included within the article.

## References

[1] Choe W. (1998). Extrarenal uptake of Tc-99m-DTPA in a case of retroperitoneal abscess causing spurious data in renal function assessment. *Annals of Nuclear Medicine*.

[2] Bihl H., Sautter-Bihl M. L., Riedasch G. (1988). Extrarenal abnormalities in Tc-99m DTPA renal perfusion studies due to hypervascularized tumors. *Clinical Nuclear Medicine*.

[3] Shih W. J., Domstad P. A., DeLand F. H. (1985). Extrarenal abnormalities in Tc-99m-DTPA renal blood flow studies. *Radiology*.

[4] Aburano T., Yokoyama K., Michigishi T., Tonami N., Hisada K. (1988). Tc-99m DTPA uptake in extramedullary plasmacytoma of the retroperitoneum. *Clinical Nuclear Medicine*.

[5] Moreno A. J., Rodriguiz A. A., Fredericks P., Kyte F. S., Turnbull G. L. (1987). Uptake of technetium-99m DTPA in a hepatic hemangioma. *Clinical Nuclear Medicine*.

[6] Goshen E., Meller I., Lantsberg S. (1991). Radionuclide imaging of soft tissue masses with Tc-99m DTPA. *Clinical Nuclear Medicine*.

[7] Roman M. R., Gruenewald S. M., Saunders C. A. (2001). The incidence of left iliac fossa uptake of 99mTc-DTPA in renal scanning. *European Journal of Nuclear Medicine*.

[8] Lee S. Y., Goh B. K., Teo M. C. (2011). Retroperitoneal liposarcomas: the experience of a tertiary Asian center. *World Journal of Surgical Oncology*.

[9] Crago A. M., Dickson M. A. (2016). Liposarcoma. *Surgical Oncology Clinics of North America*.

[10] Lee A. T. J., Thway K., Huang P. H., Jones R. L., Jones R. L. (2018). Clinical and molecular spectrum of liposarcoma. *Journal of Clinical Oncology*.

[11] Matthyssens L. E., Creytens D., Ceelen W. P. (2015). retroperitoneal liposarcoma: current insights in diagnosis and treatment. *Frontiers in Surgery*.

[12] Hornick J. L., Bosenberg M. W., Mentzel T., McMenamin M. I. N. E., Oliveira A. M., Fletcher C. D. M. (2004). Pleomorphic liposarcoma. *The American Journal of Surgical Pathology*.

[13] Wang L., Ren W., Zhou X., Sheng W., Wang J. (2013). Pleomorphic liposarcoma: a clinicopathological, immunohistochemical and molecular cytogenetic study of 32 additional cases. *Pathology International*.

[14] Evans H. L. (1979). Liposarcoma A study of 55 cases with a reassessment of its classification. *The American Journal of Surgical Pathology*.

[15] Dei Tos A. (2000). Liposarcoma: new entities and evolving concepts. *Annals of Diagnostic Pathology*.

[16] Crago A. M., Singer S. (2011). Clinical and molecular approaches to well differentiated and dedifferentiated liposarcoma. *Current Opinion in Oncology*.

[17] Singer S., Antonescu C. R., Riedel E., Brennan M. F. (2003). Histologic subtype and margin of resection predict pattern of recurrence and survival for retroperitoneal liposarcoma. *Annals of Surgery*.

[18] Dalal K. M., Kattan M. W., Antonescu C. R., Brennan M. F., Singer S. (2006). Subtype specific prognostic nomogram for patients with primary liposarcoma of the retroperitoneum, extremity, or trunk. *Annals of Surgery*.

[19] Gebhard S., Coindre J.-M., Michels J.-J. (2002). Pleomorphic liposarcoma: clinicopathologic, immunohistochemical, and follow-up analysis of 63 cases. *The American Journal of Surgical Pathology*.

[20] Beckingsale T. B., Gerrand C. H. (2009). (iii) The management of soft-tissue sarcomas. *Orthopaedics and Trauma*.

[21] Fatema C. N., Zhao S., Zhao Y. (2013). Monitoring tumor proliferative response to radiotherapy using 18F-fluorothymidine in human head and neck cancer xenograft in comparison with Ki-67. *Annals of Nuclear Medicine*.

[22] Scholzen T., Gerdes J. (2000). The Ki-67 protein: from the known and the unknown. *Journal of Cellular Physiology*.

[23] Margulis V., Shariat S. F., Ashfaq R., Sagalowsky A. I., Lotan Y. (2006). Ki-67 is an independent predictor of bladder cancer outcome in patients treated with radical cystectomy for organ-confined disease. *Clinical Cancer Research*.

[24] Xu E. W., Wang J. F., Wang Q. H. (2008). Role of Ki-67 immunostaining in diagnosis of liposarcoma. *Shanxi Medical Journal*.

